# Live imaging of micro and macro wettability variations of carbonate oil reservoirs for enhanced oil recovery and CO_2_ trapping/storage

**DOI:** 10.1038/s41598-021-04661-2

**Published:** 2022-01-24

**Authors:** A. Ivanova, A. Orekhov, S. Markovic, S. Iglauer, P. Grishin, A. Cheremisin

**Affiliations:** 1 Skolkovo Institute of Science and Technology, 121205 Moscow, Russia; 2 Moscow Institute of Physics and Technology, 141701 Dolgoprudny, Russia; 3 A.V. Shubnikov Institute of Crystallography FSRC “Crystallography and Photonics” RAS, 119333 Moscow, Russia; 4 School of Engineering, Edith Cowan University, Joondalup, 6027 Australia; 5 Western Australian School of Mines (WASM): Minerals, Energy and Chemical Engineering, Curtin University, Kensington, 6151 Australia

**Keywords:** Geochemistry, Carbon capture and storage, Geochemistry

## Abstract

Carbonate hydrocarbon reservoirs are considered as potential candidates for chemically enhanced oil recovery and for CO_2_ geological storage. However, investigation of one main controlling parameter—wettability—is usually performed by conventional integral methods at the core-scale. Moreover, literature reports show that wettability distribution may vary at the micro-scale due to the chemical heterogeneity of the reservoir and residing fluids. These differences may profoundly affect the derivation of other reservoir parameters such as relative permeability and capillary pressure, thus rendering subsequent simulations inaccurate. Here we developed an innovative approach by comparing the wettability distribution on carbonates at micro and macro-scale by combining live-imaging of controlled condensation experiments and X-ray mapping with sessile drop technique. The wettability was quantified by measuring the differences in contact angles before and after aging in palmitic, stearic and naphthenic acids. Furthermore, the influence of organic acids on wettability was examined at micro-scale, which revealed wetting heterogeneity of the surface (i.e., mixed wettability), while corresponding macro-scale measurements indicated hydrophobic wetting properties. The thickness of the adsorbed acid layer was determined, and it was correlated with the wetting properties. These findings bring into question the applicability of macro-scale data in reservoir modeling for enhanced oil recovery and geological storage of greenhouse gases.

## Introduction

Carbonate reservoirs, particularly dolomitic and calcitic limestones, contain more than half of the world’s oil and gas resources^1^. Effective exploitation of such carbonate hydrocarbon reservoirs by conventional methods, e.g., water or gas flooding, strongly depends on the wetting properties of the rock, especially in fractured low-permeability reservoirs where spontaneous imbibition of water is the main oil production mechanism^2^. With the growing interest in the application of enhanced oil recovery (EOR) techniques, for instance, injection of EOR chemicals, and the potential storage of anthropogenic CO_2_ emissions in such carbonate reservoirs, it is necessary to fundamentally understand the associated pore-scale fluid-rock interactions that control injected and displaced fluids distribution in detail.

It has been demonstrated previously that recovery efficiency from carbonates is limited due to the strong or complete oil-wetness of the rock surfaces^3–5^. This wetting condition stems from the adsorption of organic components from oil that adsorb physically or chemically onto the rock surfaces. Indeed, asphaltene and resin fractions from crude oil^6^ and organic acids (e.g., palmitic, oleic, octanoic^7^, benzoic and stearic^8,9^ acids) have a strong tendency to adsorb on chemically clean calcite surfaces, resulting in a wettability alteration from water-wet to oil-wet. Notably, carboxylic acid adsorption has been found to be higher on calcite (7.35 ± 0.15 (µmol/m^2^)) than on other minerals, such as quartz (2.76 ± 0.19 (µmol/m^2^)) or kaolinite (3.14 ± 0.25 (µmol/m^2^))^8^. It is furthermore known that organic acid content on rock surfaces is the main factor for changing the wettability (from originally water-wet) as the acid molecules form a closely packed hydrocarbon layer on the mineral surface due to chemical bonding between carboxylate groups and calcium ions^10^. Recently, it has also been shown that the presence of organic acids, particularly large organic acids such as stearic acid, on the mineral surfaces alters wettability towards more CO_2_-wet^11,12^. In this context, it has been observed that CO_2_-wet surfaces exhibit less capillary trapping capacities than hydrophilic surfaces^13–16^. Therefore, organic acid contamination in rock surfaces yields an unfavorable reduction in oil recovery and CO_2_ storage capacities of the reservoir. It is thus of vital importance to identify which organic acid (that is a component of crude oil but also exists in aquifers) has the tendency to significantly change rock wettability to enable optimal reservoir development and CO_2_ storage efficiency.

The imaging of micro and nanopore rock structures is well-established^17–21^, while direct imaging of fluid-rock interactions at the same scales, necessary for quantifying the wettability and fluids distribution, is not. Indeed, conventional laboratory methods for measuring wettability include Amott^22^, USBM (U.S. Bureau of Mines)^23^ and contact angle methods that are limited to the core or macro-scale (mm)^17^. Although these methods are standardized and commonly used, direct investigation of fluid-rock interactions is limited as these techniques provide the integral wettability of sample without accounting for surface roughness or mineral rock composition, which, however, all strongly influence multi-phase fluids flow^17^. Moreover, optical methods, such as contact angle measurements, are restricted to the resolution limit of the system that prevents detection of the contact line on curvy surfaces^24–26^. Therefore, in order to characterize wettability accurately, a combination of several different techniques, which account for micro and macro-scale surface properties, is important for better scaling and estimation of multi-phase flow parameters (e.g., capillary pressure, relative permeability). Moreover, as it was shown in Deglint et al.^17^, the wettability variations at the micro-level due to different mineralogy are very important for improved evaluation of EOR in unconventional reservoirs. Therefore, it is of crucial importance to study the different wetting states of rocks at the micro-level and compare them with the results at the macro-level.

In the present work, we thus aim to obtain deeper insight into micro-scale variations of wetting properties of carbonates caused by carboxylic acid adsorption (palmitic, stearic and naphthenic acids). In addition, the comparison of results obtained at micro and macro-scales is needed in order to assess the error that can occur in simulating the capillary pressure and relative permeability curves, fluid saturation distributions and displacement processes using only pore-scale model without taking into account the micro-scale wettability variations.

The key element of our work is the introduction of the method for the investigation of the thickness of the adsorbed layers and their influence on wetting properties at micro and macro-levels. This is achieved by combining the cryo-focused ion beam (Cryo-FIB) sample preparation with environmental scanning electron microscopy (ESEM) and scanning/transmission electron microscopy (S/TEM). Such a combination of techniques can reveal important details about the impact of organic acids on wettability, which enable accurate predictions of CO_2_ storage, H_2_ storage^27^ or oil recovery schemes^28,29^.

In this work, the experimental setup with Cryo-FIB and ESEM techniques was established in order to correlate the surface composition with its wetting properties at the micro-level. Particularly, a live-imaging ESEM microscopy approach was implemented to identify the surface areas with adsorbed organic acids layers by calculating the contact angles. The microstructure and thickness of acids layers were analyzed and calculated by using the Cryo-FIB and S/TEM experiments. Contact angles calculated at the micro-level were further correlated with ones calculated at the macro-level on the same samples before and after aging in organic acids. Therefore, this study, based on the previous advances in microscopy techniques and other imaging methods, put forward the applicability of controlled condensation at the micro-level in various scientific fields where the investigation of wetting properties is necessary.

Overall, this work improves the fundamental understanding of rock-fluid interactions and aids in the optimization of subsurface engineering processes.

## Results

### Macro-level contact angle measurements on carbonate samples before and after aging in acids

Wettability of a reservoir formation is the main parameter that strongly affects and controls oil movement throughout the pore channels^30^ and CO_2_ storage capacity and containment security^31,32^. There are several main mechanisms of preventing CO_2_ leakage to the surface, namely structural trapping (when a tight caprock serves as a barrier for CO_2_ to leak out), residual or capillary trapping (when the plum of CO_2_ splits into many bubbles, which are immobilized due to capillary forces in the porous network), mineral trapping (when CO_2_ reacts with reservoir minerals and formation brine and forms solid precipitates) and dissolution trapping (when CO_2_ dissolves in the formation brine and sinks deeper into the reservoir rocks due to gravity effect). The structural and capillary trapping mechanisms are considered to act in the first several 100 years of a storage project^11^, and thus, are of crucial importance. These two mechanisms strongly depend on capillary forces, which in turn control fluids displacement through the porous network. Notably that the effectiveness of dissolution trapping has been also found to be affected by wettability^30^.

In this context, water contact angle determines CO_2_ structural and capillary trapping capacities, and thus the total volume of residual trapped carbon dioxide^13,14^. It was shown that contact angles of θ < 50° had no effect on primary drainage, while structural CO_2_ leakage is predicted for θ > 90°^11,12,15,33^. Therefore, in this work, these angles values were used as reference values that determine the capillary and structural trapping capacities of the reservoir rocks.

**Figure 1:**
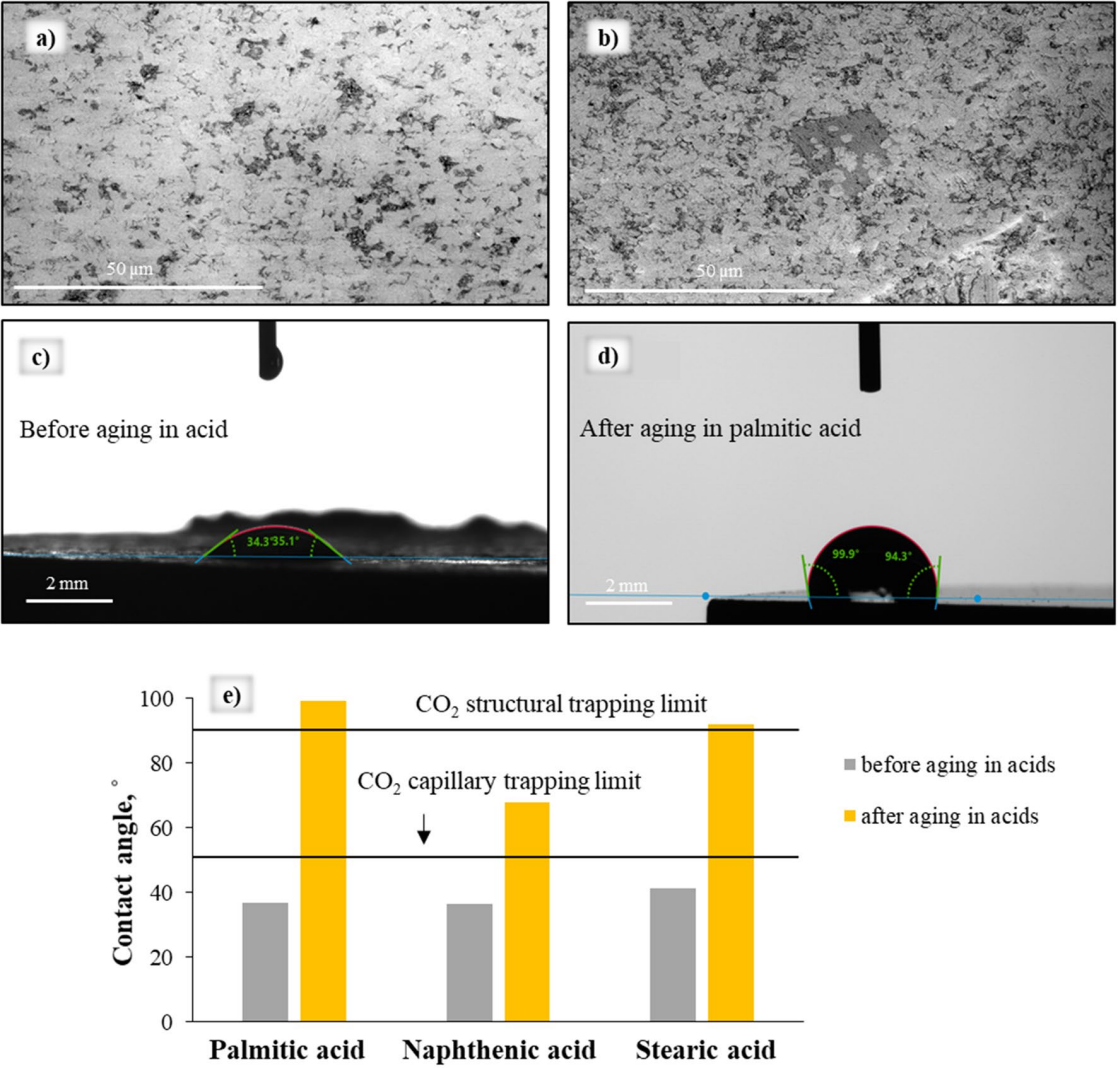
(**a**,**b**) SEM images of calcite surface before (left) and after (right) aging in palmitic acid; (**c**,**d**) examples of contact angle measurements on calcite surface before (left) and after aging (right) in palmitic acid; (**e**) Contact angle data for the samples before and after aging in different acids. Black lines indicate CO_2_ capillary and trapping limits.

In this work, the standard contact angle measurements were performed with carbonate samples before and after aging in 0.01 M solutions of palmitic, stearic and naphthenic acids (Fig. 1). It can be seen in Fig. 1c,e that the calcite (before aging) showed water-wet behavior with an average contact angle of 34.70° ± 4.06°; however, θ increased drastically after aging with organic acids, especially in case of palmitic and stearic acids, rendering the sample more hydrophobic. This phenomenon was caused by acid adsorption onto the calcite surface via covalent bonding between Ca^2+^ and COO^-^ acid groups^10^. Notably, although the adsorption mechanism is identical, the effect of different carboxylic acids on contact angle varies due to the different molecular structures of the acids.

Indeed, palmitic acid had the highest impact on wettability, shifting the contact angle to 99.36° ± 3.85°, which is similar to the effect of stearic acid (θ = 91.84° ± 2.03°). In contrast, naphthenic acid had less effect (contact angle of 67.91° ± 5.43°), which corresponds to a weakly water-wet state.

It is also clear from Fig. 1e that CO_2_ structural and capillary trapping limits are significantly affected by the adsorption of carboxylic acids. Note that the increased temperature (323 K) and pressure (25 MPa) resulted in higher contact angles (> 100°) when studying the 0.01 M concentration of stearic acid^12^.

Importantly that this effect is observed when just 0.01 M acids concentration is used, which is much lower than that found in natural oil reservoirs but probably similar to that in deep saline aquifers^34–36^.

Notably, the SEM images of the calcite surface after aging in acids (an example is shown in Fig. 1b for palmitic acid) at the micro-level (µm) suggest that the calcite surface is covered by acids irregularly. Nevertheless, the values of contact angles collected from different parts of the sample surface at the macro-level correspond to one wetting state (slightly hydrophobic).

It is thus essential to study the properties of adsorbed layers of different acids at micro-level for accurate reservoir simulation and formation CO_2_ storage capacity prediction.

### Micro-level contact angle measurements on carbonate samples before and after aging in acids

**Figure 2:**
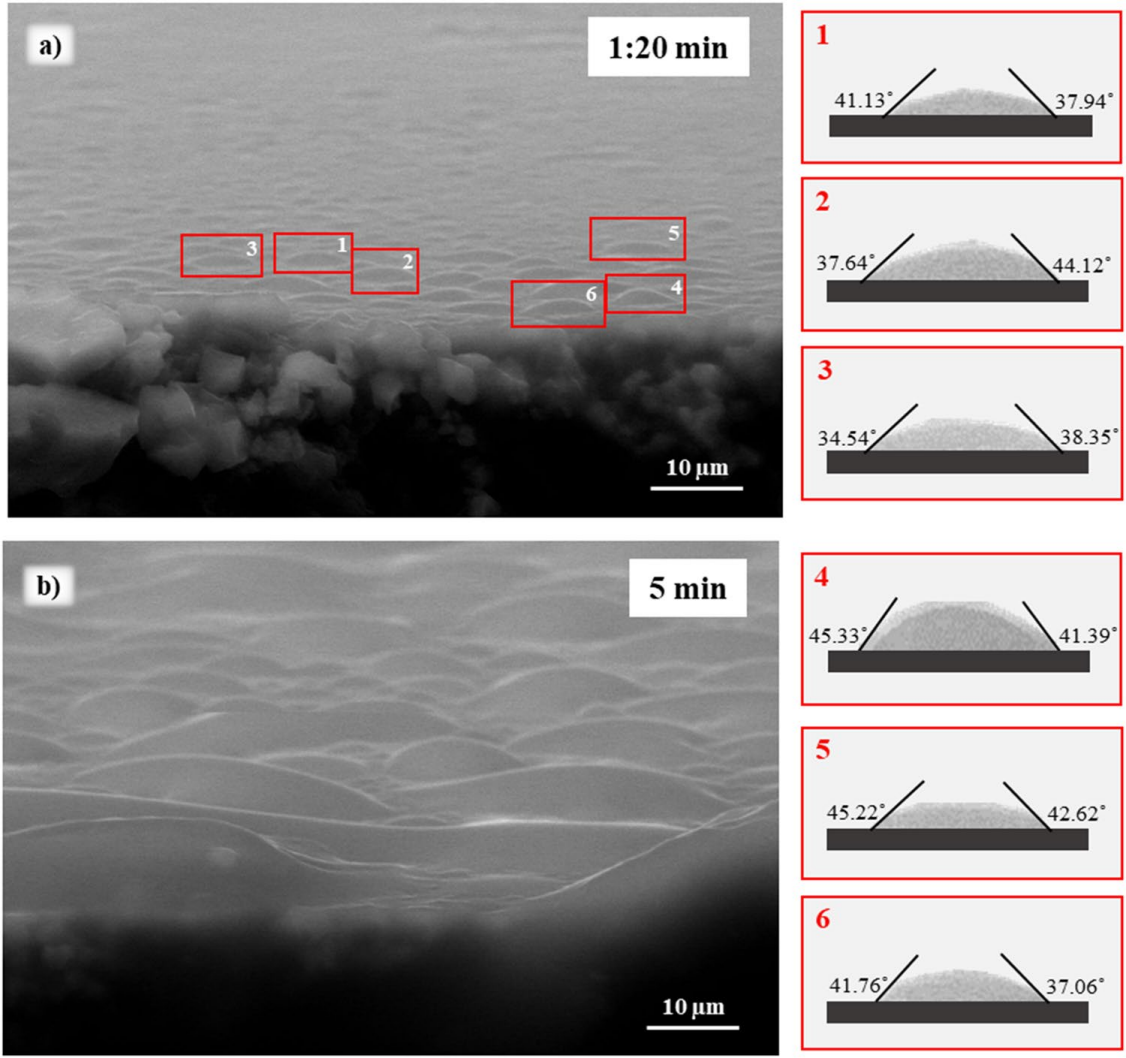
Water condensation experiments performed on calcite sample (before aging in acids) by ESEM at different times (**a**) 1:20 min and (**b**) 5 min. The chamber temperature and pressure were held at 0 °C and 899 Pa.

In order to assess wettability variations at the micro-level caused by acids adsorption, we first performed a time-lapse analysis of droplet formation on the calcite samples using ESEM. Thus, a time was selected when droplets had just appeared on the surface before they started merging to form one large drop that spreads on the entire surface. An example of a time-lapse sequence (5 min) of a condensation experiment for a calcite sample (before aging in the acid) is illustrated in Fig. 2. Micro-droplets growth started at t = 1:20 min, followed by continuous growth, resulting in a change of the three-phase contact line (Fig. 2b). Therefore, a water condensation time of 1:20 min was selected for accurate contact angle measurements.

Once the time and thermobaric conditions for water condensation were identified, the droplet profiles were extracted from the imaged regions in Fig. 2a (points 1–6) and contact angles (left and right) were calculated using the image processing software ImageJ^37^ (see “Methods” section for detailed procedure). Using this procedure, the dimensions of the extracted micro-droplet in Fig. 2a point 4 were 1.7 μm (height) and 4.07 μm (left) and 4.5 μm (right) (radii). The resulting average (left and right) contact angle is 43.36° ± 2.78°. Consequently, the average contact angle of the sample surface was calculated as 40.59° ± 3.47°.

**Figure 3:**
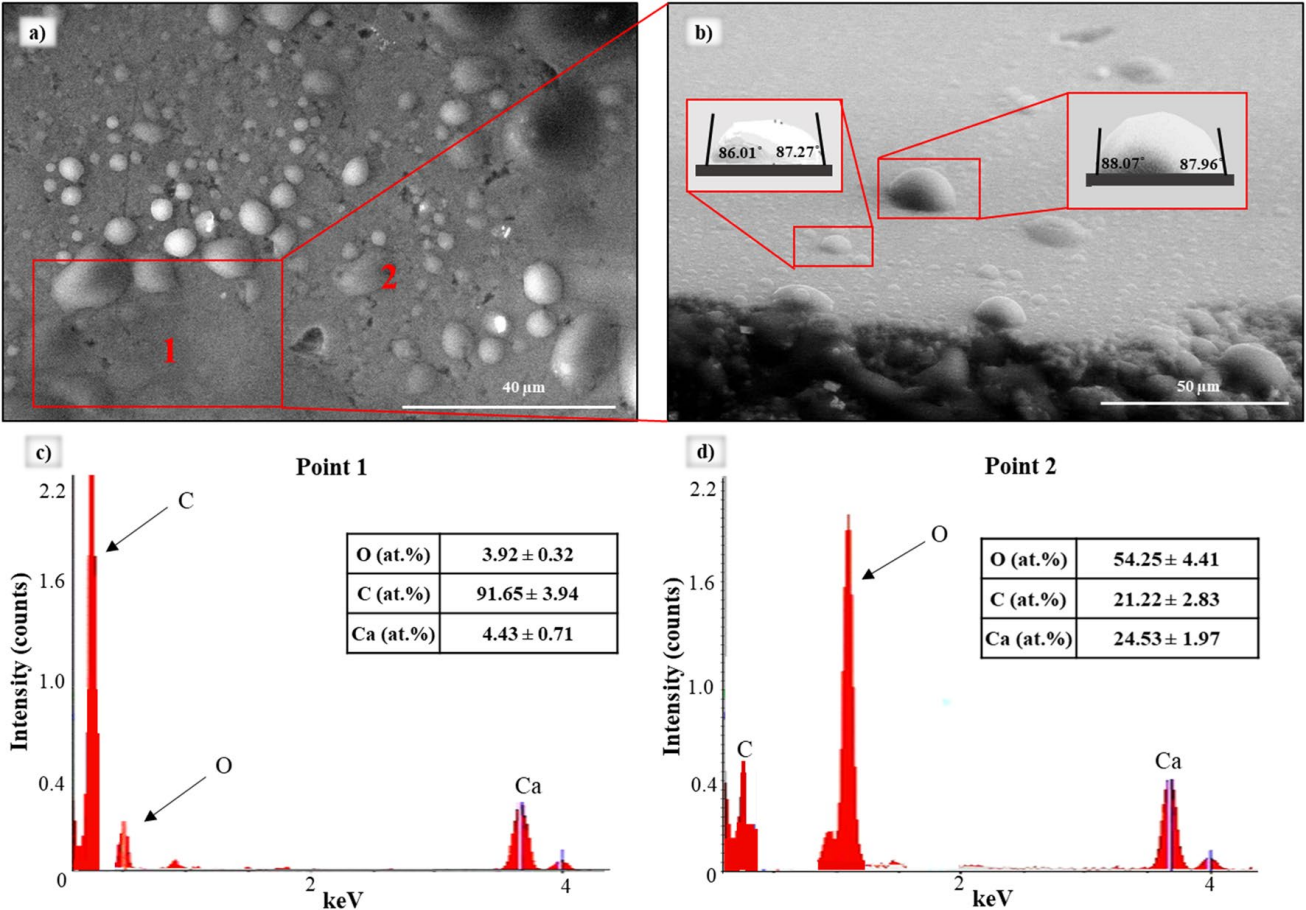
(**a**,**b**) ESEM images of a water condensation experiment showing formed water droplets on a calcite surface after aging with palmitic acid; EDX spectra of 1st point (**c**) and 2nd point (**d**) of surface, illustrating the element concentrations. Points 1 and 2 represent areas marked in (**a**) that correspond to hydrophobic and hydrophilic surface zones, respectively. The chamber temperature and pressure were held at 0 °C and 850 Pa.

Imaging and energy dispersive X-ray (EDX) analysis (of samples after aging) identified sample areas where acid layers adsorbed and their influence on micro-level wetting properties. An example is shown in Fig. 3 for the sample treated with palmitic acid. Clearly, the micro-scale wettability of the sample was variable, indicating the sample’s mixed-wettability. EDX analysis (different points were tested) revealed the elemental composition of the surface to which droplets were adhered. Consequently, the elemental composition captured in point 1 (Fig. 3a) showed a strong carbon signal (Fig. 3c)—91.65 ± 3.94 at.%; this represented the adsorbed acid layer, while in point 2, the signal showed pure calcite (proportion of elements Ca:C:O = 25:21:54 ~ 1:1:3 (Fig. 3d). Note that EDX cannot detect hydrogen atoms, and thus elemental composition data is relative.

Point 1 in Fig. 3 illustrates the sample area with intermediate wetting properties (contact angle ~ 90°), while point 2 corresponds to the hydrophilic zone of the calcite surface (contact angle < 90°). In order to analyze the adsorbed acid layers, the area marked in red in Fig. 3a was further analyzed. The shape of the droplets demonstrated that the surface was intermediate-wet with an average contact angle of 87.33° ± 0.94°, confirming the existence of additional carbon stemming from the acid.

Similar results were obtained for samples after aging in stearic or naphthenic acids, as such, the micro-scale wettability was, in fact, mixed-wet due to the irregular adsorption of acid molecules onto the rock surface. Areas with adsorbed layers of stearic and naphthenic acids showed average contact angles of 85.26° ± 1.63° and 78.35° ± 2.48°, respectively (both intermediate-wet).

### Characterization of carboxyl acid layers adsorbed on the rock surface

**Figure 4:**
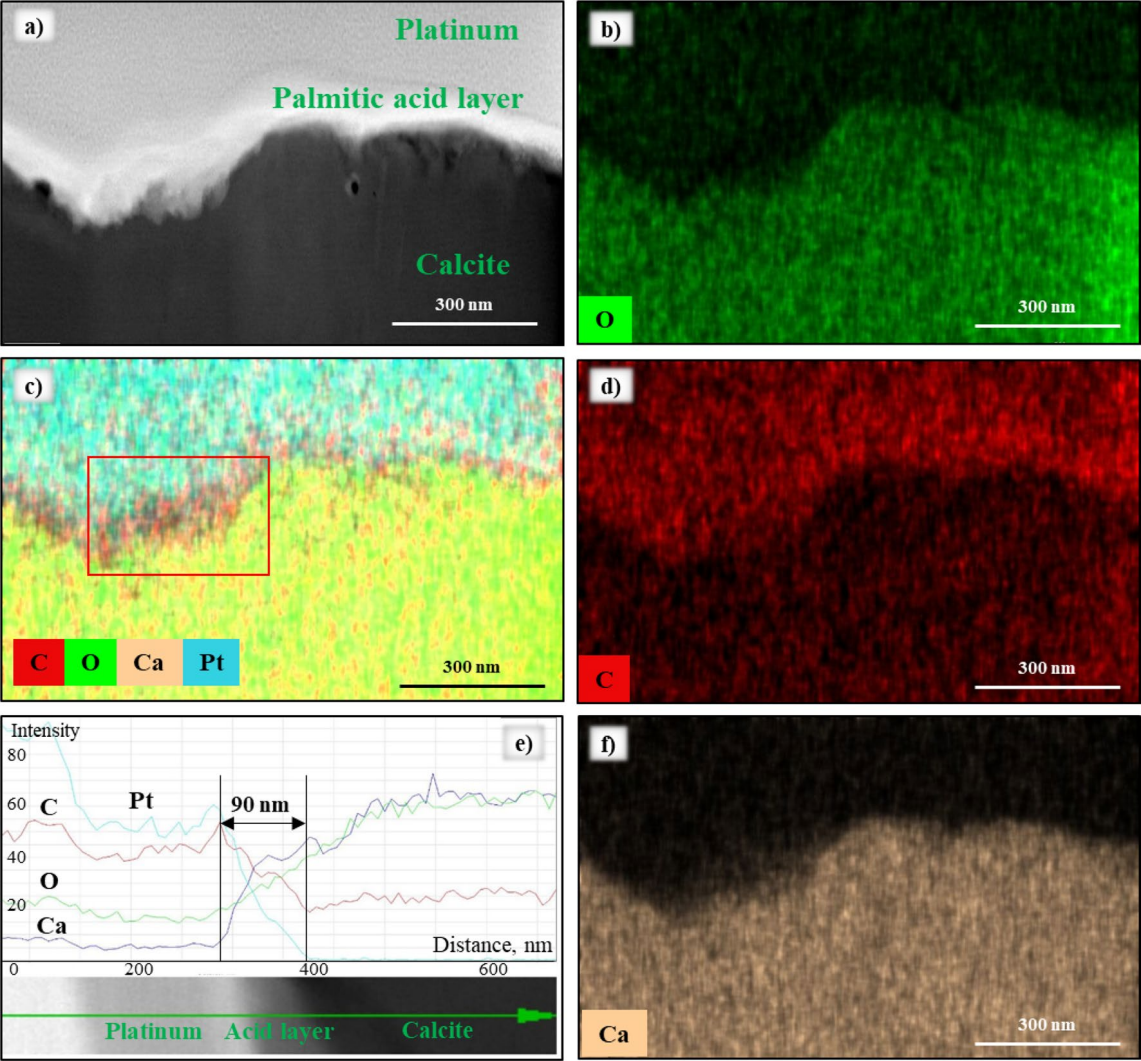
(**a**) HAADF-STEM image of the cross-section of the sample after aging in palmitic acid (note the platinum layer on top of the sample added for protection); (**b**–**d**,**f**) EDX mapping images illustrate distributions of all elements (C, O, Ca and Pt); (**e**) EDX signals of C, O, Ca and Pt observed.

**Figure 5:**
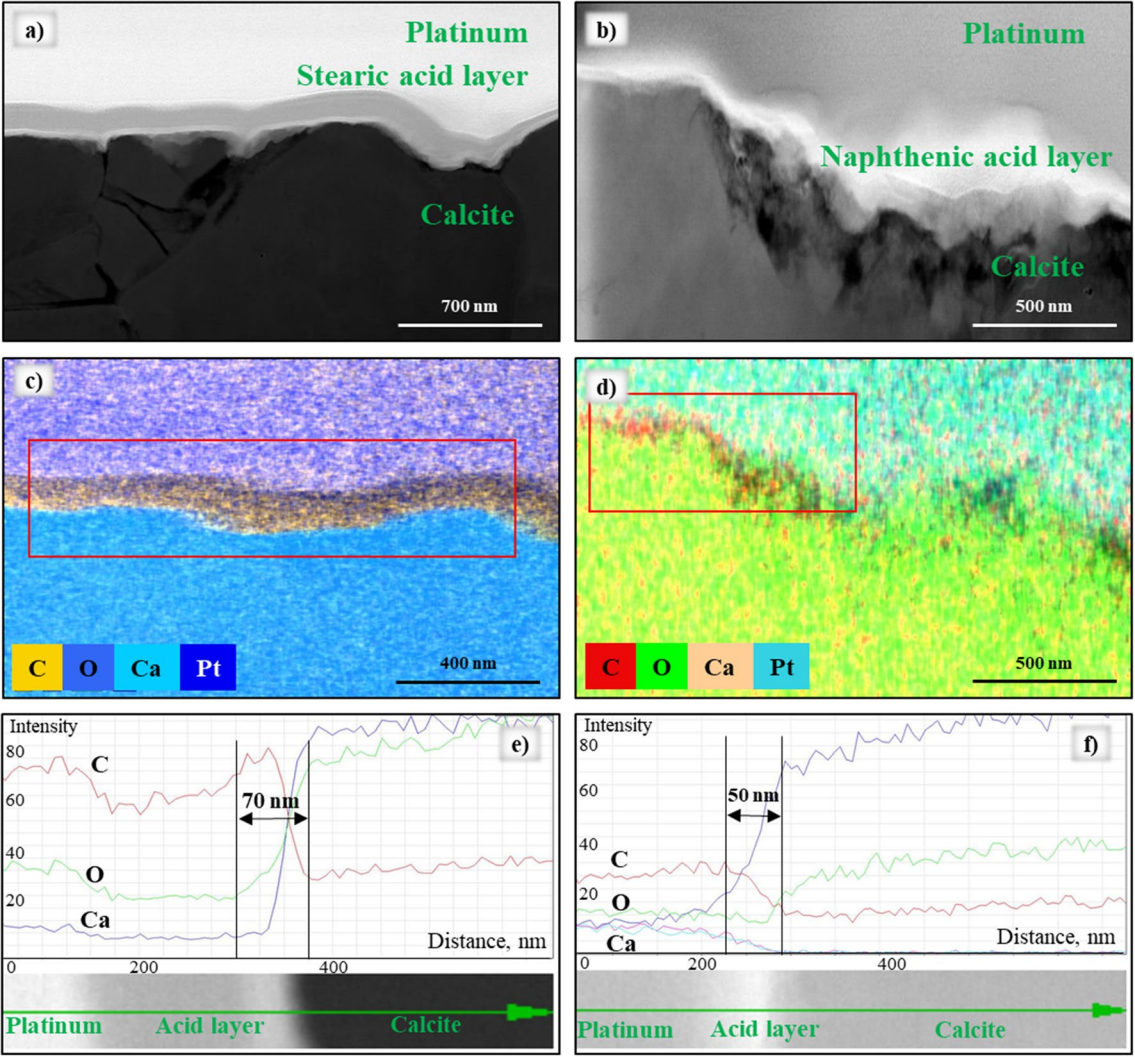
(**a**,**b**) HAADF-STEM images of cross-sections of the samples after aging in stearic and naphthenic acid (with platinum layer on the top for protection); (**c**,**d**) EDX mapping images collected illustrate distributions of all elements (C, O, Ca and Pt); (**e**,**f**) EDX signals of C, O, Ca and Pt observed.

To further examine the carboxyl acid layers on the carbonate surface, the layers were localized using the microscopic wettability results (identified by the EDX spectra of the specimen’s cross-section). Hydrophobic samples surfaces were cut by Cryo–FIB (see “Methods” section for detailed procedure). Subsequently, S/TEM and EDX analyses were performed, and the thickness and elemental composition of the layers were measured (Figs. 4 and 5). The combination of these techniques revealed fundamental wettability information at the nano-scale.

Once the cross-section specimens were cut and covered with the protection layer of platinum, the thickness was calculated by analyzing the EDX element mapping approach (Fig. 4).

The high-angle annular dark-field imaging (HAADF) and EDX mapping revealed the presence of the acids layers on the calcite surface (Figs. 4a,c and 5a–d). Clearly, the thickness of adsorbed acid layer varied, and thus the largest thickness was estimated. Owing to the changes in elemental composition spectra (Figs. 4e and 5e,f), the thickness was calculated as a difference between the highest and lowest peaks of a carbon signal. Using this criterion, the thickness of the palmitic, stearic and naphthenic acids was estimated as 85 ± 5 nm, 70 ± 8 nm and 50 ± 5 nm, respectively. Note that these layers were hydrophobic, and other hydrocarbons (e.g., from oil) can efficiently adsorb onto them and form longer and thicker layers.

The thickness of the naphthenic acid layer was the smallest among the tested acids, which is consistent with the surface wettability results (weakly water-wet with a contact angle of 67.91° ± 5.43°).

**Figure 6:**
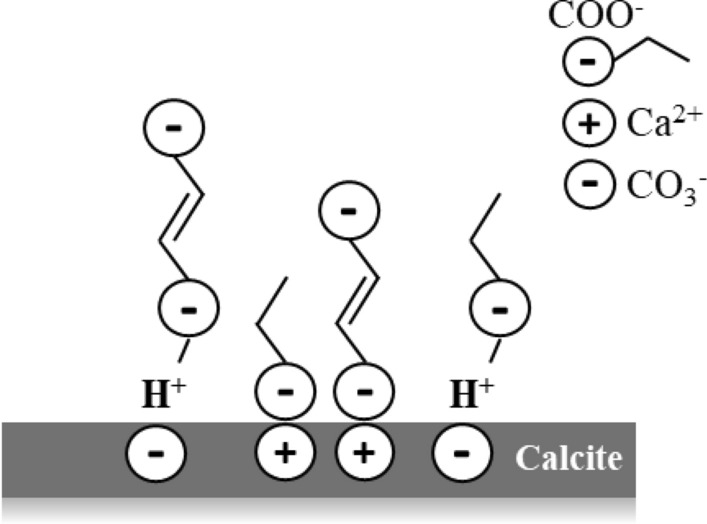
Schematic representation of carboxylic acid chemisorption onto calcite surface.

Carboxylic acids bind to a calcite surface via a condensation reaction^38^. Indeed, H^+^ ions (from the acid) approach the surface carbonate ion, whereas palmitic, stearic and naphthenic anions are attracted to the primary surface centers—Ca^2+^ ions (Fig. 6). However, due to the steric effects and different conformation of hydrocarbon chains, some surface-active centers can be blocked for acids molecules approaching. As a result, the acids adsorption is irregular on the calcite surface (Fig. 1). Furthermore, aging in naphthenic acid revealed a less significant hydrophobization effect of calcite surfaces than palmitic and stearic acids. This can be explained by taking into account the difference in the molecular structure of acids. As such, palmitic and stearic acids have liner molecular configurations, whereas naphthenic has a branched structure. Therefore, more surface area is needed for naphthenic acid molecules.

The general schematic representation of chemisorption of carboxylic acids is illustrated in Fig. 6. Thus, when the calcite surface is exposed to carboxylic acids, the first adsorbed layer is formed. Consequently, on the first layer, other acid molecules can adsorb through the hydrophobic interactions, as it is shown in Fig. 6, forming layers with different thicknesses.

## Discussion

To demonstrate the effect of carboxylic acids adsorption on the wettability of carbonate surfaces, we developed a procedure by combining various microscopic methods, including S/TEM, ESEM, Cryo-FIB and EDX mapping. Each of these techniques has strengths and limitations.

Water condensation experiments were performed through the precise controlling of chamber temperature and water saturation pressure using the Peltier stage. This technique has been carried out on different materials, including mineral^10,17^ and plant surfaces^39–42^, and thus is good-established. However, in ESEM experiments presented in this work, one of the possible errors during the sessile droplet formation is encountered due to the formation of several large droplets that cover the surface area of interest (Fig. 2). As a result, the contact line and angle between surface and droplet can be estimated inaccurately and incorrectly. In order to avoid this problem, the time-lapse experiments of water formation on surfaces should be performed each time before contact angle calculation. Note that the time of sessile droplet formation depends not only on chamber temperature and pressure but also on the nature of studied surfaces. For instance, hygroscopic minerals, i.e. inclusion of salts, can control the condensation process by restricting condensation to the areas of higher salt concentrations, and thus, not all areas can be studied^17^.

Whereas this study focused on distilled water condensation experiments (water vapor in the chamber) for the location of surface areas with adsorbed acids, the methodology can be extended to estimate the wettability variability with brine or organic liquids.

Furthermore, the roughness of the calcite surface and non-optimal micro-droplet profiles had an impact on the measurement of contact lines (between droplet and surface). We thus applied the procedure of contact angle calculation by using geometrical parameters (see Contact angle calculation at micro-scale section).

**Figure 7:**
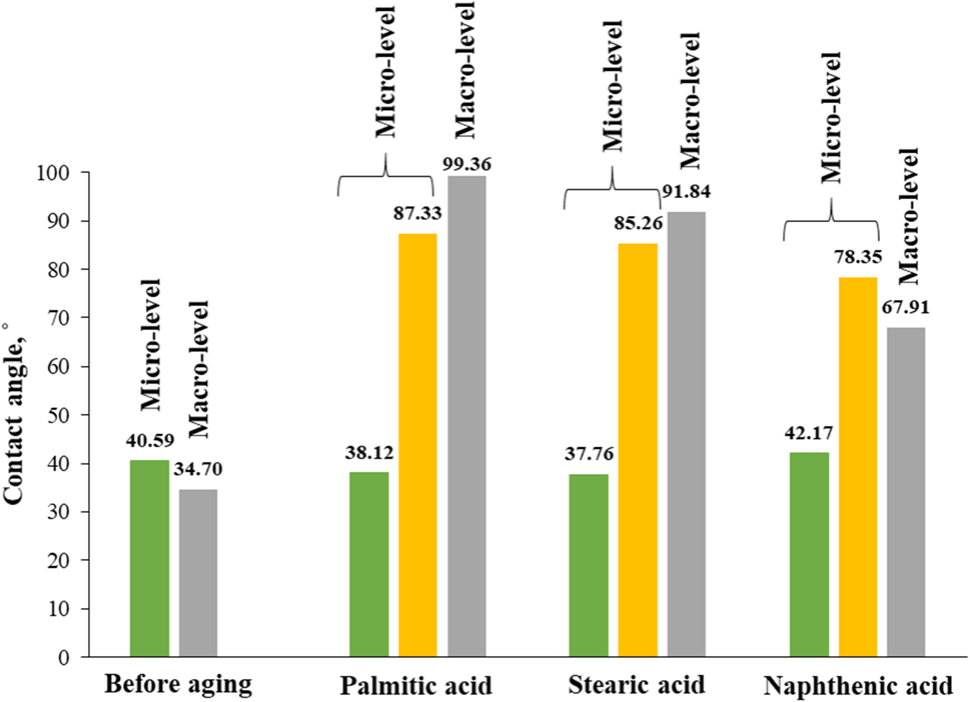
Comparison of contact angle data obtained at micro and macro scales before and after aging in palmitic, stearic and naphthenic acids.

The study of micro-scale wetting properties of samples before and after aging in acids is correlated with macroscopic surface features for the first time (Fig. 7). Figure 7 illustrates the key difference between data obtained at micro and macro scales. Clearly, the micro-wetting preferences followed the trend observed at the macro-level with a slight difference (< 15%) in contact angles. This demonstrates the hydrophilic wettability of clean surfaces (before aging), while surfaces aged in organic acids were hydrophobic. Importantly, the contact angle measurements at the micro-level provided an insight into the nano-scale wetting heterogeneity of samples aged with all acids, revealing that the surface was mixed-wet with two types of contact angle—less than 45° and more than 75° (Fig. 7). In contrast, macro-scale measurements showed that samples aged with palmitic, stearic and naphthenic acids exhibited only hydrophobic characteristics without accounting for hydrophilic zones. These results illustrate that acids adsorbed unevenly on the carbonate surface.

Core-scale wettability studies (with real reservoir cores collected from wells, where original fluids are retained) are nearly impossible, as the inherent reservoir fluids can only be preserved with special downhole retrieval processes and isothermal and isobaric transportation to the lab (note that changes in pressure or temperature may change oil and brine compositions). Typically, the wettability of such reservoir cores is reinstated to the reservoir state based on data of oil and brine composition and formation conditions. Classically, crude oil (or crude oil fractions) are used for such aging procedures, and in order to assure the accuracy of the reconstruction procedure, it is necessary to understand the nature of the initial wetting properties of rocks. Crude oil components, such as carboxylic acids and asphaltenes (in the reservoir fluid), adsorb onto the carbonate surfaces due to a condensation reaction (see above) and alter wettability towards more hydrophobic^43–45^. However, by now, and despite its importance, a serious lack of information regarding the existence, morphology, and thicknesses of these acids layers and their direct impact on wetting properties exists. Therefore, in this study, carboxylic acids (palmitic, stearic and naphthenic acids) were used as wettability modifiers as they can be found in the majority of oils around the world^7,8^. Acids layer formation on the rock surface was observed for the first time with a combined Cryo-FIB-S/TEM-EDX mapping approach across the region of interest. While wettability variations due to aging in oil are not addressed in this work, the evaluated thickness of acids layers can be used for assessing the main properties (e.g., relative permeability and capillary pressure) that affect the multi-phase fluid flow. In turn, the EOR processes and potential CO_2_ storage can be more effectively assessed.

## Conclusions

A combination of various microscopic approaches (cryogenic freezing, condensation experiments, EDX mapping) was used for measuring nano-scale wettability variations that stem from carboxylic acid adsorption onto the carbonate rock samples. The following conclusions were drawn:

1. ESEM experiments (condensation/evaporation) can be used for direct nano-level investigations of wetting variations on rock surfaces. In addition, this approach can be extended to wetting studies in other disciplines, e.g., material science or biology. In this study, the measured average nano-scale water contact angle on pure calcite was 40.59° ± 3.47°, which increased to 87.33° ± 0.94°, 85.26° ± 1.63° and 78.35° ± 2.48°, respectively, after aging in palmitic, stearic and naphthenic acids (with minor fluctuations of contact angles from left and right).
2. The ESEM experiments also illustrated that acids adsorbed on the surface unevenly, as such hydrophilic (without acids) and hydrophobic (with acids) zones were observed, and the surface exhibited mixed-wet behavior at nano-scale. In contrast, macro-scale (core-scale) experiments indicated an overall hydrophobic preference of these surfaces.
3. Cryo-FIB coupled with S/TEM successfully measured the thickness of the adsorbed acids layers adsorbed onto carbonate surface.
4. EDX mapping measured the elemental composition of the areas with different contact angle values (the carbon signal of the hydrophobic zones was higher than that of the hydrophilic zones). Moreover, EDX analysis of the specimen's cross-section allowed the determination of the thickness of the acid layers (measured thicknesses were 85 ± 5 nm, 70 ± 8 nm and 50 ± 5 nm for palmitic, stearic and naphthenic acids, respectively).
5. Results obtained herein can be used for the prediction of wetting properties of carbonate surfaces when knowing the acid number of the reservoir oil and the concentrations of carboxylic acids in it.
6. The measured acids layer thickness can aid in assessing the key meso-scale properties (e.g., relative permeability and capillary pressure) that strongly affect multi-phase fluid flow. Consequently, this work will lead to improved EOR and potential CO_2_ storage processes.

## Materials and methods

### Samples and sample preparation

**Figure 8:**
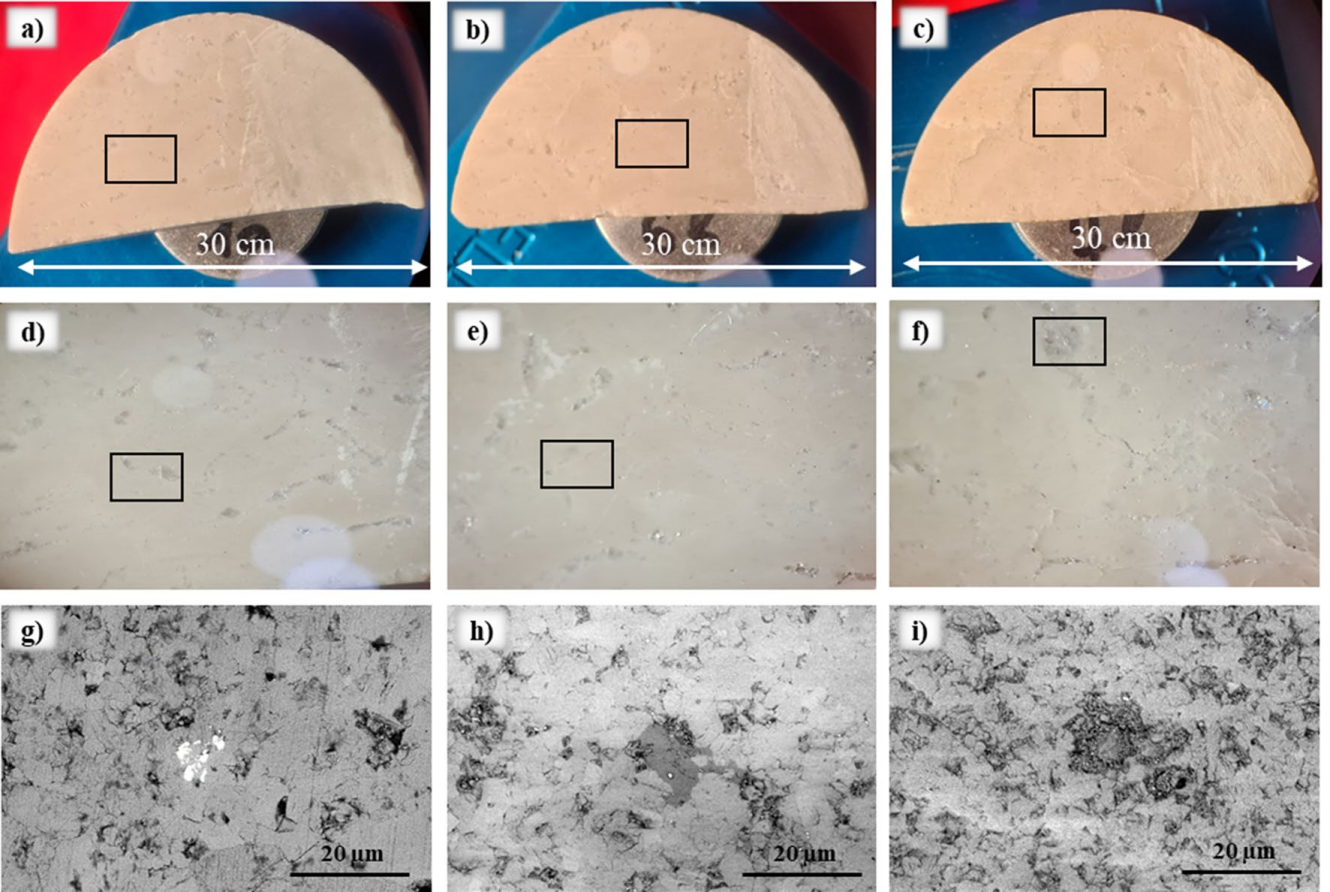
Images of the samples: (**a**) immersed into naphthenic acid solution; (**b**) immersed into palmitic acid solution; (**c**) immersed into stearic acid solution; (**d**–**f**) zoom into sample areas marked in (**a**–**c**); (**g**–**i**) SEM images of the samples areas marked in (**d**–**f**).

Core plugs were drilled from cores recovered from the water-saturated zone of an oil carbonate reservoir. The cores consisted of 99.8 wt% of calcite with minor impurities of quartz, magnetite and clay minerals. Samples were prepared for micro-and macroscopic measurements by cutting them as substrates of 5 mm thickness and 30 mm diameter (Fig. 8). Subsequently, the substrates were cleaned with deionized (DI) water to remove any impurities from the surface. Afterward, the samples were dried using compressed nitrogen for 1–3 min.

### Acid solution preparation

Three different carboxylic acids—palmitic (Sigma Aldrich, purity > 99 mol%), stearic (Sigma Aldrich, purity > 98.5 mol%) and naphthenic (Sigma Aldrich, technical grade) were used to represent typical organic acid molecules that present in crude oil and aquifers. Note that such carboxylic acids exist in deep saline aquifers^46^ as a consequence of diagenesis and biodegradation of organic matter and subsequent migration into water zones^47^.

Clearly, even very minute carboxylic acid concentrations strongly affect the wetting properties of carbonate rock surfaces^11^. Therefore, in this study, an acid concentration of 0.01M was used in all experiments, which is a good approximation of an aquifer; in an oil reservoir, organic content is substantially higher.

The palmitic, stearic and naphthenic acids were dissolved in n-decane (Sigma-Aldrich, purity > 99 mol%). The solutions were homogenized using a magnetic stirrer for one day.

### Aging procedure

In order to realistically mimic a typical reservoir formation with respect to its wetting properties, it is necessary to re-create rock surfaces that were usually exposed to formation water and hydrocarbons over geological times^12^. For this purpose, we developed the following strategy. The calcite substrates were immersed into DI water at pH = 5 at ambient conditions for 30 min. The acidity was controlled by adding drops of aqueous hydrochloric acid (HCl, Sigma Aldrich, ACS reagent, concentration 37 vol.%) and monitored by a Mettler Toledo Benchtop pH meter. This procedure enhances the adsorption rate of acids onto the substrates surfaces, and thus reproduces the years of exposure time^12^. Afterward, the samples were dried using compressed nitrogen for 1–3 min. Subsequently, the samples were immersed into solutions of palmitic, stearic and naphthenic acids of prescribed molarity and aged for 14 days at 70 °C to mimic the exposure time at reservoir temperature. Finally, the samples were dried at ambient conditions for 1 day and then used for experiments.

### Contact angle measurements at the macro-scale

The contact angle measurements on the sample substrates before and after aging with organic acids were performed by using the drop shape analysis (DSA) method, which is an image processing approach where the contact angle between the wetting phase and the surface is measured. Experimentally, a drop of wetting liquid (DI water) was spilled on the calcite substrate surrounded by air. Subsequently, a high-resolution background camera records the drop movies on the surface, whereas an image recognition algorithm recognizes the shape of the drop and the interface with a surface (baseline). The Young–Laplace contour fit technique was used and contact angle (θ) was calculated from the relationship between the interfacial tension (${\sigma }_{sl}$), surface tension of the liquid (${\sigma }_{lg}$), and the surface free energy of the substrate (${\sigma }_{sg}$):

1 $${\sigma }_{sg}={\sigma }_{sl}+{\sigma }_{lg}\cdot \mathit{cos}\theta.$$

The contact angles were captured in different positions on surfaces and calculated as a mean value of at least ten frames (measuring steps) after the equilibrium was reached. The standard deviation was calculated by determining each measured contact angle deviation relative to the mean. All measurements were carried out via a Kruss-DSA 30S analyzer at 25°C, and data was analyzed using Kruss Advance software.

Wetting properties at the macro-level were defined by the contact angles values. As such, the sample was considered hydrophilic if θ < 90°, and hydrophobic if θ > 90°. The sample showed intermediate wetting preferences if θ ~ 90°. In case the surface exhibited several wetting zones (i.e., hydrophilic and hydrophobic zones, which is relevant for the fluid dynamics), the wettability of the sample was considered mixed-wet.

### SEM

Characterization of the microstructure of the rocks was carried out using scanning electron microscopy (SEM). The sample surface was thus examined via a SEM Scios (FEI, USA) equipped with a Schottky field emission gun, a segmental solid-state detector (Z-contrast) and an EDXS system (EDAX, USA). The images were obtained in the back-scattered secondary electron (BSE) mode. The ZAF (atomic number (Z) effect; absorption (A) effect; fluorescence excitation (F) effect) correction was used as a method for uncertainty assessment that takes into account all three effects on the characteristic X-ray intensity when performing quantitative analysis.

### ESEM and Cryo-FIB measurements

Wetting properties of the aged carbonate surfaces (with acids layers) were analyzed using a SEM/FIB Versa 3D DualBeam (FEI, USA), equipped with a Quorum PP3010T Cryo-FIB/SEM Preparation System (Quorum Technologies Ltd, UK). To enable water condensation on the samples surface, the temperature was held near 0 °C (Peltier heating & cooling stages for SEM), and the chamber pressure ranged between 500 and 900 Pa (which as required to achieve the water dew point). TEM measurements were carried out using a focused ion beam at a temperature below − 140 °C. A FIB grid was placed into the vacuum chamber of the microscope on a Cryo-stage. The cross-section was prepared by Ga^+^ FIB and the sample was covered with a 2 μm Pt layer for protection (deposited by an e^−^ beam on the top of the area of interest before FIB procedure). Sequential thinning of the sample with decreasing accelerating voltages (from 30 to 2 kV) of Ga^+^ was used to minimize amorphization. Contact angles at the micro-level were defined in the same way as above (Contact angle measurement at macroscale).

### S/TEM

The cross-section of the sample was examined via a Tecnai Osiris 200 S/TEM (FEI, USA), equipped with a Schottky field emission gun, Super-X EDX detector (Bruker, USA) and CCD detector US1000 (Gatan, USA). The research was carried out at an accelerating voltage of 200 kV in bright field and dark field modes. A high angle annular dark-field (HAADF) detector (Fischione, USA) was used for obtaining Z-contrast images in the STEM mode.

### Contact angle calculation at the micro-scale

Once the conditions for droplets formulation at micro-level were found, the contact angles were calculated using the procedure described in our previous work^10^. This approach is based on measuring the height (h) and radius (r) of the droplet profiles using the Fiji platform software^37^. Hence, once geometric parameters of the droplet were calculated, the contact angle (θ) was calculated using the following equation^10^:

2 $$\theta =2\times {\mathrm{tan}}^{-1}\frac{h}{r}.$$

Note that micro-droplets were first approximated as circles in order to measure width accurately.

The contact angles were calculated as a mean value of angles from left and right sides of the droplet. The resulting value of contact angle of the surface was calculated as an average value of at least five droplets. Subsequently, the standard deviation was calculated by determining each measured contact angle deviation relative to the average.

## References

[1] Xu ZX, Li SY, Li BF, Chen DQ, Liu ZY, Li ZM (2020). A review of development methods and EOR technologies for carbonate reservoirs. Pet. Sci..

[2] Kasiri N, Bashiri A (2011). Wettability and its effects on oil recovery in fractured and conventional reservoirs. J. Pet. Sci. Eng..

[3] Brady PV, Thyne G (2016). Functional wettability in carbonate reservoirs. Energy Fuels.

[4] Alhammadi AM, AlRatrout A, Singh K, Bijeljic B, Blunt MJ (2017). In situ characterization of mixed-wettability in a reservoir rock at subsurface conditions. Sci. Rep..

[5] Shariatpanahi SF, Strand S, Austad T (2011). Initial wetting properties of carbonate oil reservoirs: Effect of the temperature and presence of sulfate in formation water. Energy Fuels.

[6] Buckley JS, Liu Y, Monsterleet S (1998). Mechanisms of wetting alteration by crude oils. SPE J..

[7] Thomas MM, Clouse JA, Longo JM (1993). Adsorption of organic compounds on carbonate minerals: 1. Model compounds and their influence on mineral wettability. Chem. Geol..

[8] Madsen L, Lind I (1998). Adsorption of carboxylic acids on reservoir minerals from organic and aqueous phase. SPE Reserv. Eval. Eng..

[9] Al-Anssari S, Wang S, Barifcani A, Lebedev M, Iglauer S (2017). Effect of temperature and SiO_2_ nanoparticle size on wettability alteration of oil-wet calcite. Fuel.

[10] Ivanova A (2019). Characterization of organic layer in oil carbonate reservoir rocks and its effect on microscale wetting properties. Sci. Rep..

[11] Iglauer S (2017). CO_2_–water–rock wettability: variability, influencing factors, and implications for CO_2_ geostorage. Acc. Chem. Res..

[12] Ali M (2019). Organic acid concentration thresholds for ageing of carbonate minerals: implications for CO_2_ trapping/storage. J. Colloid Interface Sci..

[13] Chaudhary K (2013). Pore-scale trapping of supercritical CO_2_ and the role of grain wettability and shape. Geophys. Res. Lett..

[14] Al-Menhali AS, Menke HP, Blunt MJ, Krevor SC (2016). Pore scale observations of trapped CO_2_ in mixed-wet carbonate rock: Applications to storage in oil fields. Environ. Sci. Technol..

[15] Iglauer S, Pentland CH, Busch A (2015). CO_2_ wettability of seal and reservoir rocks and the implications for carbon geo-sequestration. Water Resour. Res..

[16] Jenkins CR (2012). Safe storage and effective monitoring of CO_2_ in depleted gas fields. Proc. Natl. Acad. Sci. U. S. A..

[17] Deglint H, Clarkson CR, DeBuhr C, Ghanizadeh A (2017). Live imaging of micro-wettability experiments performed for low-permeability oil reservoirs. Sci. Rep..

[18] Schlüter S, Sheppard A, Brown K, Wildenschild D (2014). Image processing of multiphase images obtained via X-ray microtomography: A review. Water Resour. Res..

[19] Andrew M, Bijeljic B, Blunt MJ (2014). Pore-scale contact angle measurements at reservoir conditions using X-ray microtomography. Adv. Water Resour..

[20] Lin Q, Bijeljic B, Foroughi S, Berg S, Blunt MJ (2021). Pore-scale imaging of displacement patterns in an altered-wettability carbonate. Chem. Eng. Sci..

[21] Ivanova, A. et al. Microstructural imaging and characterization of organic matter presented in carbonate oil reservoirs. In *SPE EUROPEC at 81 EAGE Ann. Tech. Conf. Exhib. London, 3–6 June,* SPE-195456-MS. 10.2118/195456-MS (2019).

[22] Amott E (1959). Observations relating to the wettability of porous rock. Pet. Trans. AIMB.

[23] Donaldson EC, Thomas RD, Lorenz PB (1969). Wettability determination and its effect on recovery efficiency. J. Soc. Pet. Eng..

[24] Liimatainen V (2017). Mapping microscale wetting variations on biological and synthetic water-repellent surfaces. Nat. Commun..

[25] Srinivasan S, McKinley GH, Cohen RE (2011). Assessing the accuracy of contact angle measurements for sessile drops on liquid-repellent surfaces. Langmuir.

[26] Samuel B, Zhao H, Law KY (2011). Study of wetting and adhesion interactions between water and various polymer and superhydrophobic surfaces. J. Phys. Chem. C.

[27] Iglauer S, Ali M, Keshavarz A (2020). Hydrogen wettability of sandstone reservoirs: Implications for hydrogen geo-storage. Geophys. Res. Lett..

[28] Lin B, Cerato AB (2014). Applications of SEM and ESEM in microstructural investigation of shale-weathered expansive soils along swelling-shrinkage cycles. Eng. Geol..

[29] Robin, M., Combes, R. & Rosenberg, E. Cryo-SEM and ESEM: New techniques to investigate phase interactions within reservoir rocks. In *SPE Ann. Tech. Conf. Exhib*., Houston, Texas, 3–6 October. 10.2118/56829-MS (1999).

[30] Al-Khdheeawi EA, Vialle S, Barifcani A, Sarmadivaleh M, Iglauer S (2017). Impact of reservoir wettability and heterogeneity on CO_2_-plume migration and trapping capacity. Int. J. Greenh. Gas Control.

[31] Iglauer S, Al-Yaseri AZ, Rezaee R, Lebedev M (2015). CO_2_ wettability of caprocks: Implications for structural storage capacity and containment security. Geophys. Res. Lett..

[32] Chiquet P, Broseta D, Thibeau S (2007). Wettability alteration of caprock minerals by carbon dioxide. Geofluids.

[33] Morrow NR (1970). Physics and thermodynamics of capillary action in porous media. Ind. Eng. Chem..

[34] Kharaka YK (2009). Potential environmental issues of CO_2_ storage in deep saline aquifers: Geochemical results from the Frio-I Brine Pilot test, Texas, USA. Appl. Geochem..

[35] Thurman EM (1985). Organic Geochemistry of Natural Waters.

[36] Stalker L, Varma S, Van Gent D, Haworth J, Sharma S (2013). South West Hub: A carbon capture and storage project. Aust. J. Earth Sci..

[37] Schindelin J (2012). Fiji: An open-source platform for biological-image analysis. Nat. Methods.

[38] Mihajlovic SR (2013). Mechanism of stearic acid adsorption to calcite. Powder Technol..

[39] Tihlarikova E, Nedela V, Dordevic B (2019). In-situ preparation of plant samples in ESEM for energy dispersive X-ray microanalysis and repetitive observation in SEM and ESEM. Sci. Rep..

[40] Donald AM (2003). The use of environmental scanning electron microscopy for imaging wet and insulating materials. Nat. Mater..

[41] Paxson A, Varanasi K (2013). Self-similarity of contact line depinning from textured surfaces. Nat. Commun..

[42] Wendler J, Bown P (2013). Exceptionally well-preserved Cretaceous microfossils reveal new biomineralization styles. Nat. Commun..

[43] Gomari SR, Hamouda AA (2006). Effect of fatty acids, water composition and pH on the wettability alteration of calcite surface. J. Pet. Sci. Eng..

[44] Legens C, Toulhoat H, Cuiec L, Villieras F, Palermo T (1999). Wettability change related to adsorption of organic acids on calcite: Experimental and ab initio computational studies. J. Soc. Pet. Eng..

[45] Hakim SS (2017). Interactions of the Calcite {10.4} surface with organic compounds: Structure and behaviour at mineral—organic interfaces. Sci. Rep..

[46] Bennett PC, Siegel DE, Baedecker MJ, Hult MF (1993). Crude oil in a shallow sand and gravel aquifer—I. Hydrogeology and inorganic geochemistry. Appl. Geochem..

[47] Jones DM (2008). Crude-oil biodegradation via methanogenesis in subsurface petroleum reservoirs. Nature.

